# Diet, Inflammation, and Metabolic Complications

**DOI:** 10.3390/nu18030404

**Published:** 2026-01-26

**Authors:** Prasanth Puthanveetil

**Affiliations:** 1 Chicago College of Osteopathic Medicine, Midwestern University, Downers Grove, IL 60515, USA; pputha@midwestern.edu; 2 Department of Pharmacology, College of Graduate Studies, Midwestern University, Downers Grove, IL 60515, USA

Inflammation is mostly categorized into sterile and nonsterile inflammation [1,2,3,4,5,6,7,8,9,10]. Metabolic stress-induced systemic inflammation is a major component of sterile inflammation [1,2,11,12]. Specific diets or dietary components can represent a determinant factor in pro-inflammatory or anti-inflammatory states [1,2,11,12,13,14,15,16,17,18]. We have taken into consideration both pathogenic and sterile inflammation as a part of this discussion.

Obesity is a state of sterile inflammation and a precursor of multiple metabolic complications [19,20,21]. The work by Babicki M et al. demonstrates the importance of upgrading and revisiting health care system solutions to identify individuals with obesity in the Polish population, but the data obtained from this study are so informative that they can be applied to global perspectives [22]. Some of the key observations in this study are that subjects with obesity had elevated blood pressure and non-HDL lipoproteins along with triglycerides and glucose in a significant manner [22]. In addition, patients with obesity-related disease had inverse scores for healthy dietary choices, such as avoiding dietary harm and partaking in scheduled physical exercise that could further improve their health [22]. This work finally focuses on the importance of raising awareness around preventing obesity in the early stages, to prevent later unfavorable outcomes [22].

The next work, by Lee J H et al., also focuses on a dietary component: micronutrients [23]. This study was of a cross-sectional cohort design, with a Korean population including men and women older than fifty years [23]. There was a strong correlation between serum 25-hydroxy-Vitamin D levels and metabolic parameters. In males, lower serum 25-hydroxy-Vitamin D levels were associated with increased fat mass along with reduced muscle strength, which was also accompanied by low performance capacity in males [23]. In females, lower serum 25-hydroxy-Vitamin D levels were accompanied by higher body mass index and glycated hemoglobin (HbA1c) and inflammatory cytokines such as tumor necrosis factor-alpha [23]. There was a strong negative correlation between serum 25-hydroxy-Vitamin D levels and markers of obesity in women, which demonstrated that obesity could trigger inflammation using mediators such as TNF-α.

The next work in this Special Issue series, by Lei W et al., used a Zucker (fa/fa) rat model and found that following casein administration for 8 weeks, animals developed obesity [24]. Following the induction of obesity, animals were assigned either a casein control diet or a diet containing low or high isoflavone content for an additional 10 weeks. The consumption of a diet high in isoflavone reduced liver LPS-binding protein, in addition to the expression of inflammatory markers such as MCP-1 and TNF-α in the liver [24]. No significant changes were noted in intestinal permeability markers such as occludin, claudin 3, and zonula occludens-1 [24]. Interestingly, fecal samples from rats fed with a diet containing low and high contents of isoflavone showed significantly higher LPS concentrations compared to only CAS diet-fed rats [24]. The fecal levels of LPS were higher in isoflavone-containing diets, which highlights the influence of isoflavone in excreting toxic mediators.

The next work was completely based on an in vitro approach, using cell lines such as HT-29 cells (human female colorectal adenocarcinoma cells) and Caco-2 cells (a human cell line derived from a colon carcinoma), innate tumor cells with a high-inflammatory phenotype [25]. Here, LPS was used as an inflammation-inducing agent (a marker for pathogenic inflammation rather than sterile inflammation). LPS had a negative impact on tight junction genes such as Zona Occludin 1, Occludin, Claudin 1, and 4 [25]. These effects of LPS were reversed using onion peel extract, digested onion peel extract, quercetin, and digested quercetin. The study also revealed that all these extracts activate the protein AMPK, which, through the inhibition of NF Kappa B, reduces the cellular inflammation induced by LPS [25]. The previous study that we discussed focused on how a bioactive natural compound such as isoflavone can bring down inflammation, whereas this study compares and contrasts the source of a dietary component, onion peel (digested vs. undigested), with the naturally active compound quercetin in combating LPS-induced inflammation.

The two review articles in this Special Issue also focus on new perspectives. In their study, Givens I D et al. explore the prevalence of anemia in the Indian population and focus on multifactorial etiologies, apart from iron, that could result in anemia [26]. Based on the findings from a literature survey, the work focused on other determinants, such as a deficiency in Vitamin B_12_/folate, that could contribute to anemia [26]. The next review, by Keefe P et al., focused on comparing and contrasting two structurally close flavonoid family members [27]. This work emphasizes how apigenin and chrysin demonstrate properties of cholesterol biosynthesis inhibition and the prevention of uric acid formation or accumulation in cellular model systems [27]. Additionally, it explores how apigenin per se demonstrates the ability to enhance cellular eicosapentaenoic levels, acting as an endogenous anti-inflammatory agent, and how chrysin demonstrates an ability to inhibit pyrimidine synthesis [27]. How the overlapping and unique pharmacological properties of these two flavonoids can be made use of in treating different pathological states is yet to be determined in future studies.

Overall, the reports published in this Special Issue provide a broader view regarding the benefits of different diets and dietary components including natural products and their impact on both sterile and pathogenic inflammation.
